# A clinical trial comparing parenteral oxytetracyline and enrofloxacin on time to recovery in sheep lame with acute or chronic footrot in Kashmir, India

**DOI:** 10.1186/1746-6148-8-12

**Published:** 2012-01-31

**Authors:** J Kaler, S A Wani, I Hussain, S A Beg, M Makhdoomi, Z A Kabli, L E Green

**Affiliations:** 1 The School of Veterinary Medicine and Science, The University of Nottingham, Sutton Bonington Campus, Sutton Bonington, Leicestershire LE12 5RD, UK; 2 Bacteriology Laboratory, Division of Veterinary Microbiology and Immunology, Sher-e-Kashmir University of Agricultural Sciences and Technology of Kashmir, Shuhama (Alusteng), Srinagar 190006, Jammu and Kashmir, India; 3 School of Life Sciences, University of Warwick, Coventry CV4 7AL, UK

## Abstract

**Background:**

No clinical trials have been conducted in India on the efficacy of parenteral antibacterials to treat footrot in sheep. In addition, there are no studies worldwide on the efficacy of parenteral antibacterials to treat chronic footrot. Sixty two sheep with acute footrot and 30 sheep with chronic footrot from 7 villages in Kashmir, India were recruited into two separate trials. Sheep with acute footrot were allocated to one of three treatments using stratified random sampling: long acting parenteral oxytetracycline, long acting parenteral enrofloxacin and topical application of potassium permanganate solution (a traditional treatment used by sheep farmers in India). In a quasi pre-post intervention design, sheep with chronic footrot that had not responded to treatment with potassium permanaganate were randomly allocated to treatment with one of the two parenteral antibacterials mentioned above. Sheep with acute footrot were treated on day 0 and those with chronic footrot on days 0, 3, 6 and 9. Sheep were monitored for up to 28 days after treatment. Time to recovery from lameness and initial healing of lesions was assessed using Kaplan-Meier survival curves, nonparametric log-rank and Wilcoxon sign-rank tests.

**Results:**

There was significant correlation in recovery from lameness and presence of healing lesions in sheep with acute (r = 0.94) or chronic (r = 0.98) footrot. Sheep with acute footrot which were treated with parenteral antibacterials had a significantly more rapid recovery from lameness and had healing lesions (median = 7 days) compared with those treated with topical potassium permanganate solution (less than 50% recovered in 28 days). The median time to recovery in sheep with chronic footrot treated with either antibacterial was 17 days; this was significantly lower than the median of 75 days lame before treatment with antibacterials. The median time to recovery for both acute and chronic footrot increased as the severity of lesions increased. There was no difference in time to recovery by age, body condition score, duration lame, or presence of pus in the foot within acute and chronically affected sheep.

**Conclusions:**

We conclude that use of parenteral antibacterials to treat sheep lame with either acute or chronic footrot in India is highly effective. This is likely to improve welfare and give economic benefits to the farmers.

## Background

India ranks sixth in the world in sheep production with an estimated 61.5 million sheep [1]. Sheep farming is the main livestock industry in northern hilly areas, such as Kashmir, where it serves as a major source of income for poor, rural communities who farm sheep for mutton and wool. The system of sheep rearing in Kashmir is semi-migratory. Flocks (20-30 sheep/flock) are kept housed by owners for 4-5 months during winter. In spring, they are grouped together with other flocks under the care of a "chopan" (shepherd), who takes them to graze high altitude sub-Himalayan and Himalayan pastures. In the autumn, sheep are walked back to the valley mostly by road and are returned to their owners [2].

Footrot is an important, endemic disease of sheep in many countries including Australia, the UK, and New Zealand. Footrot is caused by *Dichelobacter nodosus *[3]. The current understanding is that *Dichelobacter nodosus *causes footrot. Other organisms, *Fusobacterium necrophorum *in particular, play an important role in facilitating the development of footrot according to some authors [3] or increasing its severity according to others [4]. Clinically, disease initially presents as an interdigital dermatitis that can progress to separation of hoof horn from the underlying sensitive tissue; there is a characteristic foul smell with all stages of disease [3]. Footrot lesions are acute when sheep have been lame for a short period of time with the above characteristic clinical signs but the foot has no chronic inflammation. Footrot lesions are chronic when in addition to the characteristic clinical signs, the hoof horn is thickened, mis-shapen or overgrown with some degree of under-running [3,4]. Sheep with chronic lesions have been reported to be lame for several weeks/months [3].

In the past few years footrot has become one of the biggest challenges for sheep farmers in Kashmir with a reported prevalence of 13 to 16% [5] and an estimated annual economic cost of approximately 37 million rupees (equivalent to approximately 8.26 million US dollars) to the sheep industry in south Kashmir [5]. Footrot spreads between sheep when they are up on the Himalayan pastures because of the warm, wet conditions [3] under foot during the day and the high stocking densities at night when sheep are grouped in paddocks to protect them from predators.

Lame sheep are not treated until they return to the villages for the winter. The most common treatment for footrot used by farmers in Kashmir is a topical application of potassium permanganate solution (authors, personal communication) to the lesion. There is no estimate of the efficacy of this method but farmers indicated that despite this treatment sheep are often lame with footrot for several months or years (personal communication). Farmers state that lame sheep are very unproductive and are often killed because of this lack of productivity.

There is evidence from clinical trials in Australia that treatment with short acting parenteral antibacterials such as penicillin and streptomycin, lincomycin, lincospectin and erythromycin [6-9] are effective against acute footrot and efficacy is improved by provision of a dry environment for 24 hours after treatment [6]. There is evidence from clinical trials in the UK that rapid recovery from footrot occurs within 3-10 days of administering long acting parenteral oxytetracycline without provision of a dry environment [10,11].

The use of parenteral antibacterials to treat footrot is not common in Kashmir and no clinical trials have been conducted in India to investigate the efficacy of such treatments for footrot. In addition, there have been no studies internationally of the efficacy of parenteral antibacterials to treat chronic footrot. The aim of the current study was to investigate the efficacy of two long acting parenteral antibacterials (oxytetracycline and enrofloxacin) on the time to recovery from both acute and chronic footrot lesions and the associated lameness in sheep in the Kashmir region of India.

## Methods

### Study design

A clinical trial was conducted on seven farms with a laboratory diagnosed history of footrot in Kashmir, north India from December 2009 to May 2010. Sheep were sourced from six private owners in the villages of Brinty and Laroo in the Anantnag district and Fatehpora, Wahidpora, Sailgam Saloora, and Shawlbugh in the Ganderbal district and finally from the Government Sheep Breeding Farm, Goabal, also in the Ganderbal district. Ninety two cross bred sheep with a minimum severity of lameness of score 2, i.e. visible nodding of the head and irregular stride but weight bearing on all four feet [12] and with active footrot (a characteristic smell, inflammation of the interdigital space and possibly under-running of hoof horn) were recruited into the trial. Their identification, age (dentition), sex, body condition score, duration of lameness at recruitment (reported by farmers) and the presence of pus in affected feet were recorded.

Footrot was categorized as chronic when there was an active lesion with a characteristic foul smell plus hyperplasia of the sole and/or wall horn with some degree of separation of the hoof from the underlying sensitive tissue and the sheep had been lame for at least 28 days. Footrot was considered acute when there was an active lesion with characteristic foul smell, interdigital inflammation with or without hoof horn separation, no hyperplasia of the sole and/or wall horn and the sheep had been lame for less than 28 days. There were 62 sheep with acute lesions and 30 sheep with chronic lesions.

The severity of footrot was scored using the system described by Egerton and Roberts [13] on a scale of 1 to 4 (scores 1-2 early stages of footrot with mild to extensive interdigital dermatitis and scores 3-4 extensive interdigital dermatitis plus under-running of hoof horn) for acute lesions and lesion scores 3-4 for chronic lesions.

Sheep with acute and chronic footrot were compared in two separate trials. In the first trial, sheep with acute footrot were randomly allocated (stratified by maximum footrot score), by drawing a coloured ball from a bag, to one of two treatment groups: long acting oxytetracycline (n = 27) (200 mg/ml, dose rate: 1 ml/10 kg intramuscular injection), long acting enrofloxacin (n = 25) (100 mg/ml, dose rate: 7.5 mg/kg intramuscular injection) and a third group was used as a control where 10 sheep (minimised for welfare reasons) were randomly allocated to receive Indian sheep farmers' traditional treatment of topical application of potassium permanganate solution. Sheep received treatment on day 0, the day of diagnosis. Researchers treated the first two groups and owner of the sheep treated the third group.

The second trial was a quasi-experiment with a pre-post treatment design [14]. Thirty sheep with chronic footrot, previously treated with local topical treatment, were stratified by maximum footrot score and then randomly allocated, by drawing a coloured ball from a bag, to one of the two antibacterial treatments (n = 13 (long acting oxytetracycline); n = 17 (long acting enrofloxacin)) as described in the first trial. Sheep were treated with antibacterials on days 0, 3, 6 and 9.

For both the trials, antibacterials were sourced from a reputable retailer and one batch number of each antibacterial type was used. The data were collected by one of 3 trained researchers who made all the observations on a farm. Sheep were inspected by researchers on days 0, 1, 2, 3, 5, 7, 14, 17, 21 and 28 and locomotion and lesion scores were recorded. A sheep recovered from lameness when it was bearing weight on all four feet with no signs of uneven posture or shortened stride (locomotion score 0) [12]. A foot was considered recovered from acute or chronic footrot when the lesion was not active: there was no foul smell or exudate and there was healing, although the tissue had not completely returned to normal.

The trial protocol was ethically approved by the Institutional Ethical Committee of Faculty of Veterinary Sciences and Animal Husbandry, SKUAST-K, Shuhama (Alusteng), Srinagar, Jammu and Kashmir, India and informed consent was obtained from the farmers.

### Data analysis

Data were stored in Microsoft Access (Microsoft) and Stata 10.0 SE (Statacorp, USA) was used for statistical analyses. Acute and chronically affected sheep were analysed separately throughout. A Wilcoxon sign-rank test was used to compare the duration of lameness in sheep with chronic footrot pre and post treatment. The correlation between time to recovery from lameness and lesions for sheep with chronic or acute footrot was assessed using Pearson's correlation coefficients (r). Kaplan-Meier survival curves and median time to recovery with 95% confidence interval (CI) were calculated for each variable and a log-rank test was used to compare equality of survivor functions by category within a variable [15]; the log-rank test for trend was conducted for ordered categories [16]. P values ≤ 0.05 were considered significant.

## Results

The distribution of acute and chronic footrot cases by farm is presented in Table 1.

**Table 1:** Details of farms recruited for the clinical trial and number of sheep with acute and chronic footrot per farm

Farm number	Village name	District	Number of acute cases	Number of chronic cases	Total number of sheep on farm
1	Fatehpora	Ganderbal	9	0	15
2	Wahidpora	Ganderbal	37	2	148
3	Saligam	Ganderbal	9	0	35
4	Shawlbugh	Ganderbal	6	3	35
5	SBF, Goabal	Ganderbal	1	5	1050
6	Brinty	Anantnag	0	9	140
7	Laroo	Anantnag	0	11	65

Total			62	30	1488

### Trial I: acute footrot

**Figure 1:**
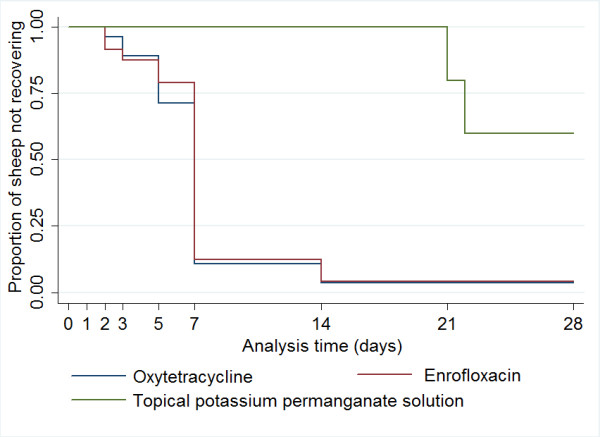
**Kaplan Meier survival curves of probability of recovery in sheep with acute footrot by type of treatment**.

There were 11 rams and 51 ewes with acute footrot. A total of 56/62 sheep had one foot affected and six sheep had 2 feet affected. All sheep, except eight, recovered from footrot and lameness within 28 days: 6/8 sheep that did not recover had been treated with topical potassium permanganate solution. Of the sheep that recovered, the median time to recovery from lesions and lameness was 7 days (95% CI: 7-14). There was a very strong positive correlation between recovery from lesions and lameness (r = 0.94, *p *< 0.05); all sheep recovered from lesions and lameness at the same observation except three sheep with acute footrot, which were lame for one week after recovery from lesions. There was no significant difference in time to recovery by the type of antibacterial used with sheep recovering in a median time of 7 days (95% CI: 7-7) with both antibacterials. However, sheep that were treated with a topical application of potassium permanganate solution were significantly less likely to recover (less than 50% sheep recovered in the trial) in comparison with injectable antibacterials (*χ*^2 ^= 24.29, *p *< 0.001; Figure 1; Table 2). Time to recovery increased significantly with the severity of lesions (stratified by type of the treatment received) (*χ*^2 trend ^= 19.72, *p *< 0.001). There was no significant difference in time to recovery by sex, farm, duration lame before treatment, age, body condition score, or presence of pus at diagnosis.

**Table 2:** Kaplan Meier estimates of median time to recovery with 95% confidence intervals (CI) for explanatory variables in sheep by acute and chronic footrot

Variable	Category	Acute footrot		Chronic footrot	
		
		N (% of all affected)	Median time to recovery (95% CI) in days	N (% of all affected)	Median time to recovery (95% CI) in days
Farm	1	9 (14)	7 (7-NE)	-	
	2	37 (60)	7 (7-7)	2 (7)	17 (14-NE)
	3	9 (14)	7 (3-NE)	-	-
	4	6 (10)	7 (7-NE)	3 (10)	19 (14-NE)
	5	1 (2)	2*	5 (16)	7 (6-NE)
	6	-	-	9 (30)	7 (7-NE)
	7	-	-	11 (37)	28 (17-28)
Age	< 4 years	34 (57)	7 (7-7)	12 (40)	14 (7-21)
	≥ 4 years	26 (43)	7 (7-7)	18 (60)	17 (7-28)
Presence of pus	Yes	6 (10)	7 (5-7)	15 (50)	8 (7-28)
	No	56 (90)	7 (7-7)	15 (50)	17 (8-21)
Body condition score	2	-	-	16 (53)	7 (7-17)
	3	52 (84)	7 (7-7)	14 (47)	21 (17-28)
	4	10 (16)	7 (2-7)	-	-
Footrot severity	Score 1 (acute)	9 (15)	3 (2-NE)	-	-
	Score 2 (acute)	44 (70)	7 (7-7)	-	-
	Score 3 (acute)	9 (15)	7 (5-14)	-	-
	Score 3 (chronic)	-	-	21 (70)	14 (7-17)
	Score 4 (chronic)	-	-	9 (30)	21 (7-NE)
No. of days lame before treatment	Up to 1 wk	41 (66)	7 (7-7)	-	-
	> 1 wk-27 days	21 (33)	7 (7-7)	-	-
	> 27 days-2 months	-	-	13 (43)	14 (7-21)
	> 2-8 months	-	-	10 (33)	17 (7-28)
	> 8-24 months	-	-	7 (24)	7 (7-NE)
Treatment	Oxytetracycline	28 (45)	7 (7-7)	12 (40)	17 (7-21)
	Enrofloxacin	24 (39)	7 (7-7)	18 (60)	14 (7-28)
	Topical treatment with potassium permanganate solution	10 (16)	^ (21-NE)	-	-

*one observation; ^less than 50% sheep recovered; *NE *not estimated

### Trial II: chronic footrot

**Figure 2:**
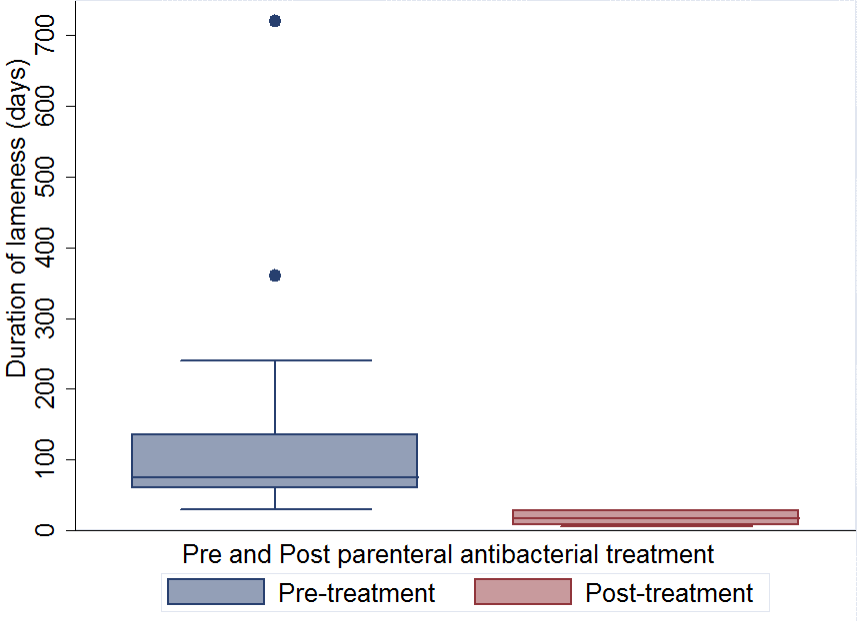
**Box plots of duration of lameness before and after treatment with parenteral antibacterials in sheep with chronic footrot**.

There were 30 ewes with chronic footrot; 27 had one foot affected and three had two feet affected. There was a significant difference (z = 4.78, *p *< 0.01) in the duration of lameness before treatment (median = 75 days (iqr (inter-quartile range): 60-135) and after parenteral antibacterial administration (median = 17 days (iqr: 7-28) (Figure 2). There was a very strong positive correlation between recovery from lesions (recovery as defined above) and lameness (r = 0.98; *p *< 0.05); there was only one sheep with chronic footrot that was lame for one week after lesions started to heal. There was no significant difference in the duration of lameness by type of antibacterial used (Table 2). All sheep, except three, recovered from chronic footrot and lameness within 28 days of parenteral antibacterial treatment. Sheep with chronic footrot had a significantly more rapid recovery on two farms (SBF, Goabal and Brinte) (Table 2) with sheep on these farms recovering in a median of 7 days compared with 17-28 days on the other three farms.

As with sheep with acute footrot, the median time to recovery from chronic footrot increased with lesion severity: with a median time to recovery of 14 d and 21 d for lesion scores 3 and 4 respectively (Table 2; *χ*^2 ^= 5.4, *p *< 0.05). There was no significant difference in time to recovery after parenteral antibacterial treatment by duration of lameness before treatment, age, body condition score, or presence of pus at diagnosis.

The time to recovery (median = 17 days) for sheep with chronic footrot was significantly higher (*χ*^2 ^= 19.48; *p *< 0.01) than for sheep with acute footrot (median = 7 days). Sheep with chronic footrot had lower body condition scores (range 2 to 3) than sheep with acute footrot (range 3 to 4). A significantly higher (50%) percentage of sheep with chronic footrot had pus in the foot compared with sheep with acute footrot (12%).

No sheep became lame again during the 28 day observation period in both the trials.

## Discussion

This is the first clinical trial, to the authors' knowledge, to demonstrate recovery from chronic footrot and its associated lameness. The rapid time to recovery with parenteral antibacterials is remarkable given that these sheep had been lame from 28 days to two years. It does highlight that systemic treatment with 12 days of therapeutic level of antibacterials is highly effective against this bacterial disease, even for chronic lesions where sheep have a persistent footrot lesion with hyperplasia and misshapen hoof horn [3,4]. Although sheep with chronic lesions recovered from lameness and had no active footrot lesion in the current study, we do not know whether or when feet with hyperplastic horn returned to normal physical conformation. Hoof horn damage takes several months to resolve [3,17] and the follow up period for the current trial was only 28 days, hence no information could be collected on improvement in physical conformation. There is evidence that sheep with poor foot shape are more likely to improve their foot quality after treatment with parenteral antibacterials [17].

There were no time parallel controls in the trial with sheep with chronic footrot, instead, sheep acted as their own control in a pre and post quasi experimental design. These sheep had been lame for such a long time and given local treatments so it seemed unethical to not give these sheep the parenteral antibacterials that are the best known treatment for footrot [10]. Quasi experimental designs are widely used in medical and social research where it is unethical to either withhold a treatment that has known efficacy or where there is a need to intervene quickly e.g. to prevent spread of infectious disease [14]. There is a possibility of confounding by factors that could have been different between the two time periods (pre and post treatment) such as climate. Climate has been shown to influence both the severity and spread of footrot [3]. However, given that there was a wide range of time in the duration of lameness it is highly unlikely that recovery after treatment with antibacterials occurred for any other reason than the treatment given.

In Kashmir, there are many challenges faced by farmers in managing footrot. Due to practical constraints, sheep that develop footrot whilst on Himalayan pastures are not treated until they return to the valley. They walk a substantial distance to return home, usually on hard roads that thicken and harden the hoof horn. Once home, lame sheep are typically treated as described in the introduction. Parenteral antibacterials are generally not used; farmers have been told they are ineffective and there are always concerns about the price and quality of pharmaceuticals (authors, personal communication). The results from the current trial indicate that if farmers use a course of four long acting injections of a relatively inexpensive well sourced antibacterial, there will be welfare benefits to the sheep and economic gain to the owners because once they are not lame and in pain [18] these sheep will gain body condition and have improved conception rates and lambing percentages [11]. There are sheep both in India and other countries with chronic footrot--either through neglect or inappropriate treatment. There is every reason to consider that the treatment presented in the current study would be effective in resolving footrot in these sheep.

As importantly, sheep with acute footrot recovered in a median 7 days after treatment with antibacterials, similar to other studies [10,11]. However, sheep that received the traditional treatment of topical potassium permanganate solution were lame for longer with only 4 recovering within 28 days suggesting that this is an ineffective treatment. A solution of potassium permanganate is generally made at home by using the crystals that are readily available and cheap (authors' personal communications). Its use is common in these communities to disinfect water and as a general antiseptic for human skin conditions at concentration of 0.01% [19]. Factors such as the deep seated location of *D. nodous *in footrot lesions [3] and/or an incorrect concentration of the solution could have contributed to low cure rates. It is possible that for very mild cases of interdigital dermatitis topical application leads to recovery. The lesions that received topical treatment in the current study ranged from moderate interdigital damage to some separation. There was no information collected on the strength of solution used by the farmers.

There was no significant difference between long acting oxytetracycline and enrofloxacin on time to recovery from footrot in the current trial. Enrofloxacin is a third generation fluoroquinolone, a broad-spectrum bactericidal antibacterial that is commonly used in veterinary and human medicine in India [20]. It was used in the current trial because of its common use and accessibility in India. Given the global concern with the development of antibacterial resistance with flouroquinolones [21,22] and the fact that the long acting oxytetracycline was equally effective, we consider that long acting oxytetracycline, which is rarely used in human medicine, should be used to treat sheep with acute or chronic footrot in India.

In the current trial, sheep were followed for up to 10 days after recovery or a maximum of 28 days after treatment. No sheep treated with parenteral antibacterials became lame again during this period; however, we do not know whether footrot recurred after the end of the trial. Sheep with acute footrot with damaged feet and poor foot integrity are more likely to get footrot again, although this is less likely if they have been given parenteral antibacterials [17].

There was a significant association between lesion severity and time to recovery from footrot in both acute and chronic cases, with severe lesions with extensive interdigital dermatitis and separation of horn taking longer to heal. This is in contrast to other trials [9,10] which reported no link between severity of lesions and time to recovery. However, in Kaler et al. [10] and Jordan et al. [9] the majority of the sheep (> 94%) had the most severe lesion score (score 4), in addition, Kaler et al. [10] used a different system to classify lesion severity which might have affected the results obtained.

Sheep in the current trial were scored by three trained observers (with one observer making all the observations per farm); this could have led to some observer bias. However, the same observer was used to score all the sheep and follow up observations to minimize observer variability. Unlike sheep with acute lesions where there was no difference in recovery by farm, sheep with chronic lesions had a significantly shorter time to recovery on two farms compared with the other three farms. The sheep on each of these farms were scored by two different observers. The fact that these observers also scored acute cases and that there was no differences in time to recovery by farm suggests that this is less likely to be observer bias unless observers were only biased when scoring recovery from chronic lesions. Sheep on these two farms had lesion score severities 3 and 4 and duration of lameness from 3 months to 2 years which suggests the rapid recovery was unlikely to be a confounding effect of severity of footrot or duration of lameness. There is a possibility that the treated chronic cases on these two farms had better foot integrity than other farms which led to rapid recovery [3,17], however, no information on foot integrity was recorded in this trial to investigate this.

## Conclusions

We conclude that parenteral antibacterials are an effective treatment for both acute and chronic footrot and associated lameness in sheep in India. Their use to treat sheep lame with footrot in Kashmir would vastly improve welfare of these sheep with considerable economic benefit to the farmers.

## Authors' contributions

JK contributed to the design of the trial, performed the data analysis and drafted the manuscript. LEG contributed to the design of the trial, advised on data analysis and interpretation and oversaw finalising the manuscript. SAW, IH, SAB, MM, ZAK conducted the clinical trials at the seven farms, collected the data and contributed to the preparation of the manuscript. All authors read and approved the final manuscript.
